# Virtual Nuclear Envelope Breakdown and Its Regulators in Fission Yeast Meiosis

**DOI:** 10.3389/fcell.2016.00005

**Published:** 2016-02-02

**Authors:** Haruhiko Asakawa, Hui-Ju Yang, Yasushi Hiraoka, Tokuko Haraguchi

**Affiliations:** ^1^Graduate School of Frontier Biosciences, Osaka UniversitySuita, Japan; ^2^Cell Biology Group, Advanced ICT Research Institute Kobe, National Institute of Information and Communications TechnologyKobe, Japan; ^3^Graduate School of Science, Department of Biology, Osaka UniversityToyonaka, Japan

**Keywords:** closed mitosis, fission yeast, meiosis, nuclear envelope breakdown, RanGAP1, Nup132

## Abstract

Ran, a small GTPase, is required for the spindle formation and nuclear envelope (NE) formation. After NE breakdown (NEBD) during mitosis in metazoan cells, the Ran-GTP gradient across the NE is lost and Ran-GTP becomes concentrated around chromatin, thus affecting the stability of microtubules and promoting the assembly of spindle microtubules and segregation of chromosomes. Mitosis in which chromosomes are segregated subsequent to NEBD is called “open mitosis.” In contrast, many fungi undergo a process termed “closed mitosis” in which chromosome segregation and spindle formation occur without NEBD. Although the fission yeast *Schizosaccharomyces pombe* undergoes a closed mitosis, it exhibits a short period during meiosis (anaphase of the second meiosis; called “anaphase II”) when nuclear and cytoplasmic proteins are mixed in the presence of intact NE and nuclear pore complexes (NPC). This “virtual” nuclear envelope breakdown (vNEBD) involves changes in the localization of RanGAP1, an activator of Ran-GTP hydrolysis. Recently, Nup132, a component of the structural core Nup107-160 subcomplex of the NPC, has been shown to be involved in the maintenance of the nuclear cytoplasmic barrier in yeast meiosis. In this review, we highlight the possible roles of RanGAP1 and Nup132 in vNEBD and discuss the biological significance of vNEBD in *S. pombe* meiosis.

## Introduction

In eukaryotic cells, the nucleus is enclosed by a nuclear envelope (NE) in interphase. The NE is a double membrane structure which separates the nucleoplasm from the cytoplasm. Macromolecules are transported between the nucleus and the cytoplasm across the NE through nuclear pores which are formed by large protein complexes called nuclear pore complexes (NPCs; Reichelt et al., 1990). The NPC has an eight-fold rotational symmetry structure (Hinshaw et al., 1992; Akey and Radermacher, 1993; Kiseleva et al., 1996) and is composed of ~30 kinds of proteins called nucleoporins (Yang et al., 1998). Nucleoporins can be classified into several groups according to their localization and function: membrane-integrated nucleoporins, cytoplasmic filaments, scaffold subcomplexes, adaptor subcomplexes, central channels, and nuclear baskets (Rout et al., 2000; Cronshaw et al., 2002; Mans et al., 2004; Osmani et al., 2006; Alber et al., 2007; DeGrasse et al., 2009; Iwamoto et al., 2009; Tamura et al., 2010; Asakawa et al., 2014). Distinct nucleoporin subclasses are involved in Ran-dependent nucleocytoplasmic transport as described below.

**Figure 1:**
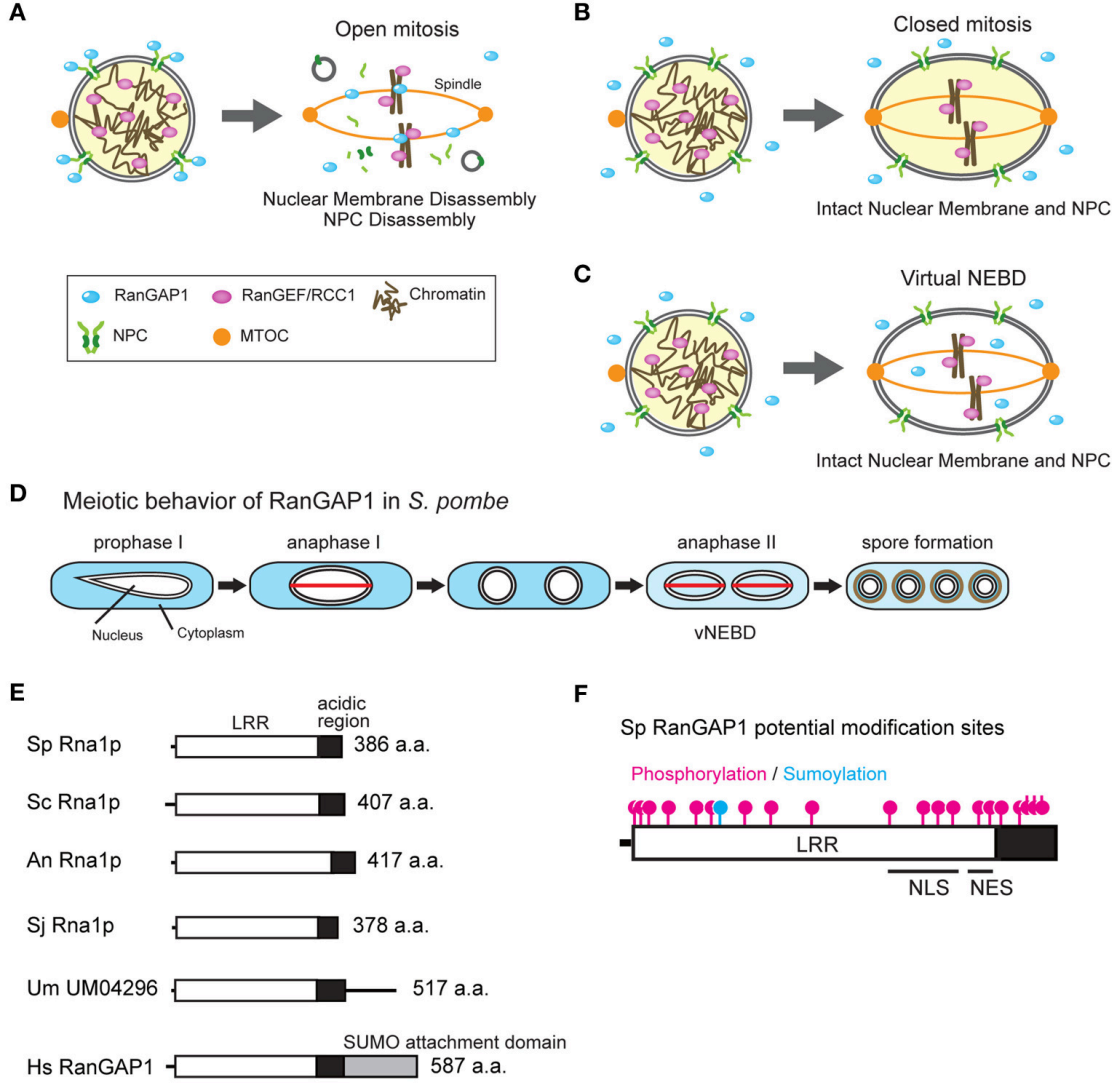
**(A-C)** Open and closed mitosis, and virtual nuclear envelope breakdown. **(A)** Interphase nucleus (left) and open mitosis (right) in metazoa. In interphase, the RanGAP1 is associated with the nuclear pore complex (NPC) whereas the RanGEF/RCC1 is associated with chromatin, maintaining the Ran-GTP gradient. In open mitosis, the nuclear membrane and NPCs are disassembled at the beginning of mitosis and nuclear and cytoplasmic proteins are mixed together. Mitotic spindle is elongated from both poles of the microtubule organizing center (MTOC) and capture kinetochores. SUMO-conjugated RanGAP1 associates with mitotic kinetochores and spindle microtubules and the remaining RanGAP1 diffuses throughout the cell. The disassembled nuclear membranes (shown as gray circles) are absorbed into the ER (Ellenberg et al., 1997; Yang et al., 1997). **(B)** Interphase nucleus (left) and closed mitosis (right) in fungi. In interphase, the Ran-GTP gradient is maintained by cytoplasmic RanGAP1 and chromatin-bound RanGEF/RCC1. In closed mitosis, the nuclear membrane and the NPCs remain intact, and the Ran-GTP gradient across the NE is maintained. Mitotic spindle is formed in the nucleus between the MTOCs which are penetrated into the NE. **(C)** Virtual nuclear envelope breakdown. During meiosis II in *S. pombe*, both the nuclear membrane and NPCs remain intact but RanGAP1 enters the nucleus, resulting in the abolishment of the Ran-GTP gradient. **(D)** Behavior of RanGAP1 during meiosis. RanGAP1 (blue) remains in the cytoplasm during the entire meiotic process, except anaphase II. During anaphase II, RanGAP1 diffuses throughout the cell. The red lines shown in anaphase I and II represent spindles. **(E)** Domain structure of RanGAP1. Molecular domains of RanGAP1 in eukaryotes are shown. Leucine-rich repeat (LRR; white box), acidic region (black box), and SUMO attachment domain (gray box) are indicated. Sp, *S. pombe*; Sc, *S. cerevisiae*; An, *A. nidulans*; Sj, *S. japonicus*; Um, *U. maydis*; Hs, *Homo sapiens*. *U. maydis* homolog UM04296 contains an additional amino acid sequence after the acidic region; however, the function of this sequence is unknown. **(F)** Potential post-translational modification sites, NLS, and NES in *S. pombe* RanGAP1. Magenta and blue indicate potential phosphorylation and sumoylation sites, respectively, that have been predicted using the sequence of *S. pombe* RanGAP1 (Asakawa et al., 2011). The putative NLS and NES have been predicted by Feng et al. (1999).

Nucleocytoplasmic transport depends on the activity of Ran, a small GTPase (Moore and Blobel, 1993; Moroianu and Blobel, 1995). Ran exists in two forms and is bound to either GTP or GDP (Scheffzek et al., 1995; Vetter et al., 1999). The GTP-bound form of Ran (Ran-GTP) is predominantly nuclear but also exists at low levels in the cytoplasm (Bischoff and Ponstingl, 1991b). This gradient of Ran-GTP between the nucleus and the cytoplasm establishes and maintains the direction of nucleocytoplasmic transport (Izaurralde et al., 1997; Nachury and Weis, 1999; Kaláb et al., 2006). The gradient of Ran-GTP is generated based on the localization of two important proteins called Ran GTPase activating protein 1 (RanGAP1) and Ran guanine nucleotide exchange factor (RanGEF/RCC1; Izaurralde et al., 1997; Nachury and Weis, 1999; Kaláb et al., 2006). RanGAP1, which converts Ran-GTP to Ran-GDP, is cytoplasmic (Bischoff et al., 1994; Becker et al., 1995; Seewald et al., 2002). In humans, RanGAP1 has been shown to require sumoylation within its C-terminal domain by Nup358/RanBP2 for localization to NPCs (Matunis et al., 1996, 1998; Mahajan et al., 1997; Saitoh et al., 1997). Nup358/RanBP2, a cytoplasmic filament nucleoporin, is a small ubiquitin-like modifier (SUMO) E3 ligase complex that catalyzes SUMO post-translational modification to target proteins (Pichler et al., 2002). In this way, RanGAP1 activity is concentrated at the cytoplasmic side of the NPC. On the other hand, RanGEF/RCC1, which converts Ran-GDP to Ran-GTP, is a chromatin-associated protein and its activity is concentrated in the nucleus (Ohtsubo et al., 1987, 1989; Bischoff and Ponstingl, 1991a,b; Figure 1A). This biased localization of RanGAP1 and RanGEF/RCC1 generates the gradient of Ran-GTP between the nucleus and the cytoplasm.

For nucleocytoplasmic transport, most proteins and RNA are carried by transport receptors of the importin β superfamily such as importin β or exportin. These proteins are structurally similar (reviewed in Conti et al., 2006), but are distinguished by their binding properties to cargos upon binding to Ran-GTP. Importin β releases its cargo when it binds to Ran-GTP in the nucleus and binds to its cargo in the cytoplasm where Ran-GTP is hydrolyzed to GDP. In contrast, exportin binds to its cargo in the nucleus when it binds to Ran-GTP and releases its cargo in the cytoplasm where Ran-GTP is hydrolyzed to GDP (reviewed in Cook et al., 2007). In mammals, there are 14–20 members of the importin β/exportin superfamily (reviewed in Kimura and Imamoto, 2014). Thus, the Ran-GTP gradient plays a critical role in determining the direction of nucleocytoplasmic transport.

## Variation of mitosis

During mitosis in metazoa, NE breakdown (NEBD) occurs (open mitosis) (reviewed in Güttinger et al., 2009; Figure 1A). NEBD causes diffusion of nuclear and cytoplasmic molecules within the entire cell, resulting in loss of the compartmentalization of guanine nucleotide-bound forms of Ran on either side of the NE. Simultaneously, SUMO-conjugated RanGAP1 associates with mitotic kinetochores and spindle microtubules and the remaining RanGAP1 diffuses throughout the cell (Joseph et al., 2002, 2004). In contrast, RanGEF/RCC1 remains associated with chromatin and thus the Ran-GTP gradient is shifted and remained only close regions around chromosomes (Kaláb et al., 2006). In metazoa, NEBD is essential for chromosome segregation because mitotic spindles are formed from cytosolic microtubule organizing centers (MTOCs) or centrosomes that exist at opposite ends of the cell and function to capture kinetochores after NEBD. In contrast, many fungi undergo mitosis without NEBD (closed mitosis) (Heath, 1980; Figure 1B). During closed mitosis, the spindle is formed in the nucleus between the spindle pole bodies (equivalent to centrosomes in metazoa) which are embedded in the NE, and chromosomes segregate without NEBD (Figure 1B). This manner of mitosis is observed in many fungi including yeasts.

In addition to closed mitosis, recent research has revealed unique types of mitoses in some fungal species (De Souza et al., 2004). In the filamentous fungus *Aspergillus nidulans*, “semi-open” mitosis has been reported (De Souza et al., 2004). During mitosis in *A. nidulans*, the NE remains largely intact but NPCs undergo partial disassembly. In this type of mitosis, peripheral nucleoporins that form cytoplasmic filaments, adaptor subcomplexes, central channels, and nuclear baskets are disassembled, leaving structural core nucleoporins such as the Nup107-160 subcomplex and membrane-integrated nucleoporins in the NE (De Souza et al., 2004; Osmani et al., 2006). Disassembly of these nucleoporins disrupts the compartmentalization of the nucleus and alters the localization of *A. nidulans* RanGAP1 during mitosis, resulting in the relocalization of RanGAP1, which is conventionally localized only to the cytoplasm during interphase, to both the cytoplasm and the nucleus during mitosis (De Souza et al., 2004). Nucleoporin disassembly in *A. nidulans* requires NIMA and CDK kinases. Particularly, the kinase activity of NIMA is associated with the phosphorylation of nucleoporin Nup98 in *A. nidulans* (De Souza et al., 2004). This is similar to NPC disassembly observed during open mitosis in metazoans where Nup98 is phosphorylated by NIMA and CDK kinases. The hypo-phosphorylated mutant of Nup98 delays NPC disassembly (Laurell et al., 2011).

The fission yeast *Schizosaccharomyces japonicus* exhibits another type of semi-open mitosis in which the nuclear envelope ruptures in anaphase (Aoki et al., 2011; Yam et al., 2011; Gu et al., 2012). In this organism, the mitotic spindle is formed in the nucleus and undergoes elongation in the limited nuclear space to form a bent spindle (Yam et al., 2011). The NE of *S. japonicus* is ruptured in the medial region when the nucleus elongates during anaphase (Aoki et al., 2011; Yam et al., 2011). The semi-open mitosis in *S. japonicus* involves APC/C activity that induces the degradation of Oar2, a 3-oxoacyl-[acyl-carrier-protein] reductase (Aoki et al., 2013). Oar2 is a conserved protein that elongates fatty acids and makes phospholipids, a source for cellular membranes (Schneider et al., 1997). The degradation of Oar2 likely decreases the sources required for membrane synthesis and leads to the breakage of the NE in *S. japonicus* during mitosis.

The corn smut basidiomycete fungus *Ustilago maydis* undergoes “open” mitosis, in which the nuclear envelope is disassembled upon entry into mitosis. This organism shows growth dimorphism of yeast and hyphae forms. In the yeast form of *U. maydis*, the nucleus elongates and moves from the mother cell to the daughter cell. When the nucleus reaches the daughter cell, the NE ruptures at the leading edge of the stretched nucleus and recedes into the mother cell (Straube et al., 2005). During this process, NPCs disassemble and disperse into the cytoplasm similar to that observed in organisms undergoing open mitosis (Theisen et al., 2008). These data indicate that these special modes of mitosis in *A. nidulans, U. maydis*, and *S. japonicus* require partial disruption of the NE integrity or alteration of NPC composition.

## vNEBD and its regulators

### RanGAP1

A special mechanism that reduces the Ran-GTP gradient during chromosome segregation in fission yeast *S. pombe* has been reported. *S. pombe* undergoes closed mitosis and meiosis I during which nuclear transport activity is maintained. However, during anaphase in meiosis II (i.e., anaphase II), nuclear and cytoplasmic molecules are mixed similar to open mitosis. Interestingly, both the NE and NPCs are maintained in this phase and thus this phenomenon is called “virtual” NEBD (vNEBD; Figure 1C; Arai et al., 2010; Asakawa et al., 2010).

vNEBD coincides with altered RanGAP1 localization from the cytoplasm to the nucleus (see “anaphase II” in Figure 1D). RCC1/RanGEF remains associated with chromatin throughout anaphase II (Arai et al., 2010; Asakawa et al., 2010). Ectopic expression of RanGAP1 fused with a nuclear localization signal (NLS) results in the mislocalization of GFP-NLS from the nucleus to the cytoplasm (Asakawa et al., 2010). These observations suggest that altered RanGAP1 localization abolishes the Ran-GTP gradient at the nucleus. Mobile characteristics of RanGAP1 in *S. pombe* may be due to its protein structure (Figure 1E). In humans, RanGAP1 is sumoylated at the C-terminal and is targeted to the cytoplasmic filaments of NPCs (Matunis et al., 1996; Mahajan et al., 1997; Saitoh et al., 1997). In organisms undergoing closed mitosis, including *S. pombe*, RanGAP1 does not have the C-terminal sumoylation domain (Figure 1E). In fact, *S. pombe* RanGAP1 is a cytoplasmic protein and is not associated with the NE (Figure 1C; Arai et al., 2010; Asakawa et al., 2010). On the other hand, *S. pombe* RanGAP1 contains several residues that may undergo post-translational modifications, including phosphorylation and N-terminal sumoylation (Figure 1F). Amino acid substitution of some of these residues in endogenous RanGAP1 results in cell death, thus these residues are important for the function of RanGAP1 (Asakawa et al., 2011). However, it is unknown whether these residues are required for vNEBD.

RanGAP1 of budding yeast *Saccharomyces cerevisiae* contains an NLS and a nuclear export signal (NES) that regulate its localization (Feng et al., 1999). These NLS and NES sequences are also conserved in *S. pombe* RanGAP1 (Figure 1F; Feng et al., 1999). Although it is unclear whether these sequences function as NLS and NES, it has been observed that *S. pombe* RanGAP1 binds to histone H3 and is required for heterochromatin assembly during vegetative growth (Nishijima et al., 2006), suggesting that RanGAP1, at least in part, is actively transported into the nucleus. This may explain how alteration of RanGAP1 localization actively occurs during vNEBD.

### Nucleoporins

**Figure 2:**
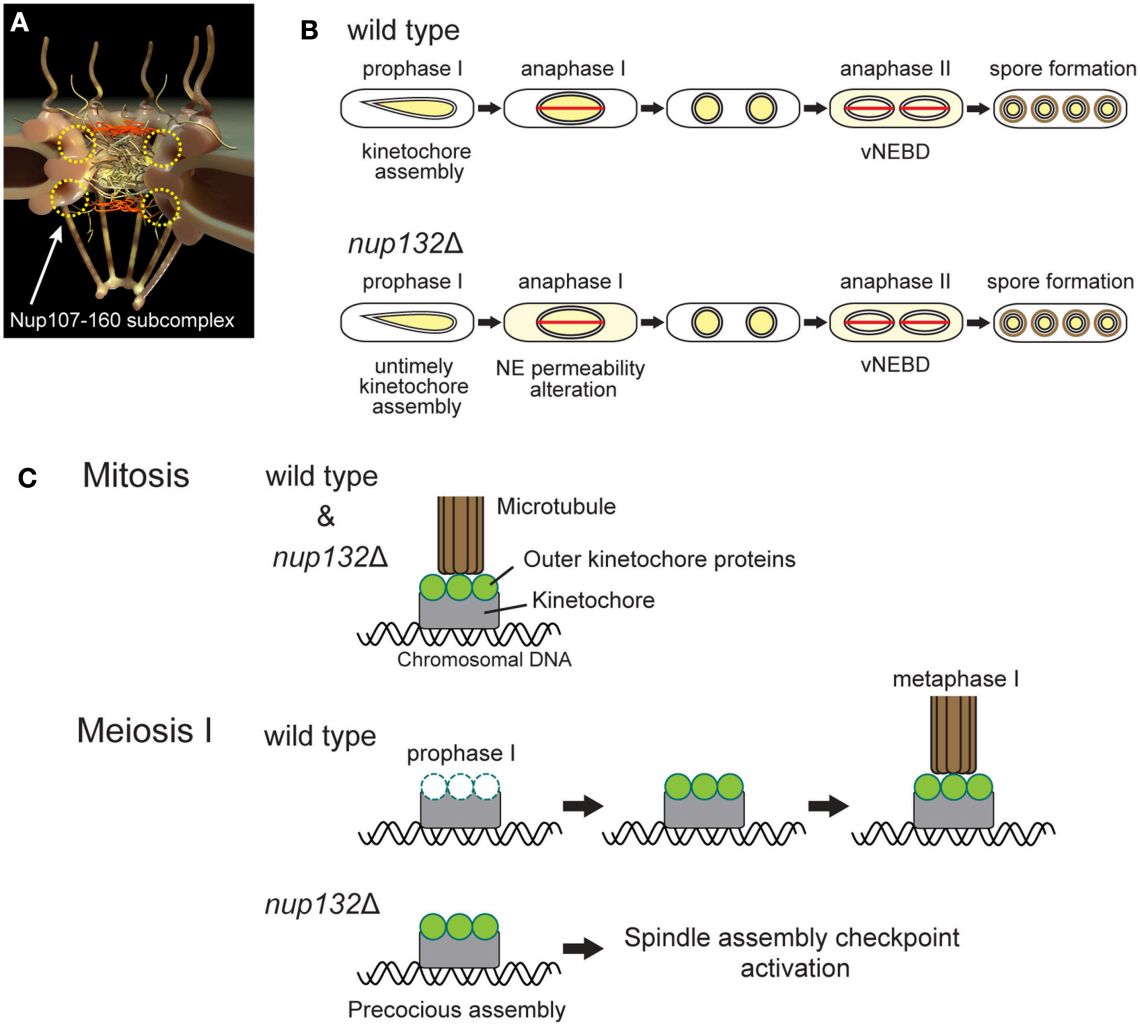
**Meiotic phenotypes of *nup132* deletion mutant cells. (A)** NPC structure. The yellow circles indicate the putative positions of the Nup107-160 subcomplex. **(B)** Comparison of nuclear protein behavior between wild-type and *nup132* mutant (*nup132*Δ) cells. Yellow indicates the localization site of nuclear proteins. The red lines shown in anaphase I and II represent spindles. **(C)** Attachment of kinetochores and microtubules during mitosis (upper panel) and meiosis I (lower two panels). During mitosis in wild-type and *nup132*Δ cells, kinetochores (gray) are connected to microtubules (brown) through outer kinetochore proteins (green). During meiotic prophase I in wild-type cells, the outer kinetochore proteins (green) disassemble and reassemble at the kinetochores to form normal kinetochores for meiosis I. In contrast, *nup132*Δ cells show a precocious assembly of the outer kinetochore proteins (green).

A recent study showed that there are eight non-essential nucleoporins, the mutation of which is associated with defective production of spores per ascus (Asakawa et al., 2014), suggesting their involvement in vNEBD. The phenotype of the nucleoporin gene *nup132* disruption mutant has been reported recently (Yang et al., 2015). *S. pombe* Nup132 is a homolog of Nup133, which is a component of a conserved Nup107-160 subcomplex of NPCs (Lutzmann et al., 2002; Harel et al., 2003; Walther et al., 2003; Baï et al., 2004; Osmani et al., 2006; DeGrasse et al., 2009; Tamura et al., 2010; Figure 2A). The *S. pombe nup132* mutant shows two major meiotic phenotypes (Figure 2B): (1) untimely kinetochore assembly during prophase of meiosis I (prophase I) and (2) loss of barrier function of the NE during anaphase of meiosis I (anaphase I; Yang et al., 2015). Notably, in this mutant, overall nuclear permeability during meiotic prophase I is not affected.

Our discovery raises the question of how Nup132 regulates both kinetochore assembly and NE permeability during meiosis I in *S. pombe*. Interestingly, the metazoan Nup107-160 subcomplex is known to re-localize to the kinetochores upon NEBD and functions in kinetochore-microtubule attachment (Loïodice et al., 2004; Orjalo et al., 2006; Zuccolo et al., 2007; Mishra et al., 2010), hinting at relocalization of Nup132 to the kinetochore during *S. pombe* meiosis I. In addition, Nup133, a human homolog of *S. pombe* Nup132, is required for proper localization of RanGAP1 at the kinetochore in human cells (Zuccolo et al., 2007). However, because of closed meiosis I in yeast, Nup132 remains at the NE (Asakawa et al., 2010), and there is no evidence of Nup132 relocalization to the kinetochores. The effect of Nup132 on kinetochores in the absence of NEBD should be investigated in future studies.

Inadequate kinetochore-microtubule attachment in the *nup132* mutant activates the spindle assembly checkpoint and delays meiosis I progression (Figure 2C; Yang et al., 2015). The duration of meiosis II is also prolonged in this mutant, but this prolongation is independent of the spindle assembly checkpoint (Yang et al., 2015). Alternatively, loss of barrier function of the NE during anaphase I might induce defects in subsequent nuclear division during meiosis II. This is consistent with the overexpression of Sid1, a component of the septation initiation network (SIN) signaling pathway, that induces the precocious leakage of nuclear proteins through the NE during meiosis I, leading to defects in chromosome segregation (Arai et al., 2010). These results highlight the importance of temporal control of vNEBD during anaphase II.

As mentioned above, Nup132 depletion results in the loss of barrier function of the NE during anaphase I (Yang et al., 2015), suggesting that altered permeability of the NE depends on the function of only one nucleoporin during meiosis. This is supported by the fact that depletion of *S. cerevisiae* Nup133 results in Ran being uniformly localized in the cell rather than being enriched in the nucleus (Gao et al., 2003). How does Nup132 regulate the permeability or barrier function of the NE? One explanation is that in both *S. pombe* and *S. cerevisiae*, Nup132/Nup133 is required for the uniform distribution of NPCs along the NE (Doye et al., 1994; Li et al., 1995; Pemberton et al., 1995; Baï et al., 2004) and that unevenly distributed NPCs hamper nucleocytoplasmic transport (Steinberg et al., 2012). A second explanation is that Nup132 depletion may disrupt the structural integrity of NPCs because Nup132 is a component of the Nup107-160 subcomplex that forms the NPC scaffold structure. Thus, Nup132 depletion may lead to the partial disassembly of NPCs and induce “semi-open” meiosis I. A third explanation is that Nup132 depletion induces early vNEBD during anaphase I through yet unknown mechanisms involved in cell cycle progression. Identification of GFP-fused nucleoporins and NE proteins in the *nup132* mutant will help determine whether changes in nuclear permeability during anaphase I involve NPC disassembly or NE rupture.

Functional alterations in Nup132 or other nucleoporins may change the barrier function of the NE without inducing NPC disassembly. Post-translational modifications in nucleoporins may alter the barrier function of NPCs. In mammals, nucleoporin Nup50 is phosphorylated by ERK kinase, which in turn changes the affinity of importin β to NPCs (Kosako et al., 2009). Thus, the barrier function of NPCs during meiosis in *S. pombe* may be regulated by phosphorylation or other post-translational modifications of nucleoporins. Further studies are required to elucidate the role of post-translational modifications of Nup132 or other nucleoporins in the regulation of barrier function of NPCs and vNEBD during meiosis in *S. pombe*.

### Cdc2 kinase

The timing of vNEBD corresponds to meiosis II during which *S. pombe* cells produce spores, and a correlation of vNEBD with spore formation has been established (Arai et al., 2010; Asakawa et al., 2010). Spore formation and meiotic nuclear division are coordinately regulated by Cdc2 kinase (Nakaseko et al., 1984; Grallert and Sipiczki, 1991). Similar to that observed during mitosis, Cdc2 kinase activity increases at the onset of meiosis I and dramatically decreases upon the completion of meiosis I. Transition from meiosis I to meiosis II also requires an increase in Cdc2 activity. The *mes1* mutant, which is deficient in blocking the degradation of cyclin Cdc13 (Izawa et al., 2005), arrests before meiosis II due to insufficient re-activation of Cdc2 for starting meiosis II (Izawa et al., 2005; Kimata et al., 2008, 2011). Moreover, the *mes1* mutant shows no vNEBD (Arai et al., 2010; Asakawa et al., 2010) and is defective in spore formation (Shimoda et al., 1985; Izawa et al., 2005), suggesting a correlation between them. In contrast, the *tws1* mutant, a meiosis-specific allelic mutant of Cdc2 (MacNeill et al., 1991), does not undergo meiosis II and forms two diploid spores after meiosis I (Nakaseko et al., 1984). This mutant allele is thought to affect interactions between Cdc2 and its binding proteins (MacNeill et al., 1991). In the *tws1* mutant, nuclear proteins diffuse to the cytoplasm and RanGAP1 localizes to the nucleus during meiosis I (Arai et al., 2010; Asakawa et al., 2010), suggesting that hypophosphorylation of a Cdc2 substrate during meiosis I may possibly drive vNEBD. It is unclear whether Cdc2 regulates vNEBD through nucleoporins and/or RanGAP1 or through lipid metabolism pathways similar to that observed during semi-open mitosis in *S. japonicus* (Aoki et al., 2013).

## Significance of vNEBD

vNEBD occurs specifically during anaphase II and results in the diffusion of nuclear proteins into the cytoplasm and cytoplasmic proteins into the nucleus, which is similar to that observed during open mitosis. vNEBD may allow cytoplasmic proteins to function in the nucleus during or after anaphase II. Occurrence of vNEBD is correlated with spore formation (Arai et al., 2010; Asakawa et al., 2010). *S. pombe* produces spores under nutritionally starved conditions. These spores are metabolically inactive until they experience growth-favorable conditions (Shimoda and Nakamura, 2004). Because of their dormancy, it is tempting to think that the nuclei of these spores are transcriptionally silent. NPCs are suggested to be involved in gene regulation and chromatin organization (Therizols et al., 2006; Zuccolo et al., 2007; Van de Vosse et al., 2013; Pascual-Garcia and Capelson, 2014; Breuer and Ohkura, 2015; Yang et al., 2015). Based on these data and the fact that nuclear RanGAP1 triggers heterochromatin formation in *S. pombe* (Nishijima et al., 2006), we propose that functional alterations in NPCs that accompany vNEBD enable global chromatin reorganization for generating dormant nuclei.

## Conclusion

The phenomenon of vNEBD suggests the importance of “opening” the gate between the nucleus and cytoplasm during meiosis. Nucleoporins and RanGAP1 may play key roles during open meiosis without physically breaking down the NE in *S. pombe*. This provides an example in which the regulated barrier function of the NE plays an important role for regulating meiotic processes in eukaryotes.

### Conflict of interest statement

The authors declare that the research was conducted in the absence of any commercial or financial relationships that could be construed as a potential conflict of interest.
